# Advancing Tuberculosis Vaccination: Immunological Insights and Emerging Strategies for Improved Protection

**DOI:** 10.3390/vaccines14100896

**Published:** 2026-10-13

**Authors:** Zhicheng Zhang, Crystal Sanchez, Derek Baker, Jeries Bawardi, Victoria Renteria, Sheralyn Sanchez, Subramanian Dhandayuthapani, Jessica Chacon

**Affiliations:** 1 Foster School of Medicine, Texas Tech University Health Sciences Center, El Paso, TX 79905, USA; zhiczhan@ttep.edu (Z.Z.); derebake@ttep.edu (D.B.); jbawardi@ttep.edu (J.B.); victoren@ttep.edu (V.R.); sheralyn.sanchez@ttep.edu (S.S.); 2 Department of Medical Education, Foster School of Medicine, Texas Tech University Health Sciences Center, El Paso, TX 79905, USA; 3 Department of Medicine and Oncology, University of Texas Rio Grande Valley, Edinburg, TX 78541, USA

**Keywords:** tuberculosis, vaccination, Bacille Calmette–Guérin

## Abstract

Tuberculosis (TB) remains one of the leading causes of infectious disease mortality worldwide despite widespread Bacille Calmette-Guérin (BCG) vaccination and decades of control efforts. The limited and variable efficacy of current vaccination strategies, particularly against adult pulmonary disease, highlights the need for a deeper understanding of the host immune mechanisms that determine protection against *Mycobacterium tuberculosis*. While traditional approaches to TB vaccine development have largely focused on pathogen-specific immunity and clinical efficacy, emerging evidence suggests that successful TB control may depend equally on the strategic modulation of host immune responses. This narrative review examines the immunological basis of TB vaccination, emphasizing how contemporary vaccine platforms reshape both innate and adaptive immunity. We discuss the roles of trained immunity, T-cell function, immune memory, and mucosal immune responses in mediating protection, as well as the mechanisms by which *M. tuberculosis* evades immune surveillance. The review further explores next-generation vaccine technologies, including protein-adjuvant, viral-vectored, live-attenuated, and mRNA-based vaccines, highlighting their distinct immunological targets. Particular attention is given to mucosal immunity, systems vaccinology, and the ongoing search for reliable correlates of protection, which collectively offer new opportunities to improve vaccine design and evaluation. Finally, we examine host-directed immunomodulatory approaches, immune-engineering strategies, and precision vaccinology as emerging paradigms for TB prevention. By reframing TB vaccines as instruments for immune reprogramming rather than solely pathogen-specific interventions, this review provides an integrated perspective on how advances in immunology may accelerate progress toward global TB control and the goals of the WHO End TB Strategy.

## 1. Introduction

### 1.1. Global Burden of Tuberculosis and the Need for Improved Prevention

Despite a century of Bacille Calmette-Guérin (BCG) vaccination, tuberculosis (TB) remains one of humanity’s deadliest infectious diseases, highlighting that effective TB control is not merely a microbiological challenge, but fundamentally an immunological one. Tuberculosis, caused by *Mycobacterium tuberculosis* (Mtb), remains a major global health threat, causing an estimated 10.7 million new cases and approximately 1.23 million deaths worldwide in 2024, despite the availability of effective antimicrobial therapy and BCG vaccination [1]. The disease disproportionately affects low- and middle-income countries the most, where socioeconomic disparities, HIV coinfection, malnutrition, and limited access to healthcare continue to sustain transmission and mortality. Although global TB control efforts have reduced disease burden in some regions, progress toward the World Health Organization (WHO) End TB Strategy targets remains insufficient, underscoring the urgent need for more effective preventive strategies.

### 1.2. Clinical Role and Limitations of BCG Vaccination

BCG has been the cornerstone of TB prevention since 1921, providing substantial protection against severe childhood diseases, including miliary TB and tuberculous meningitis [2]. However, protection against transmissible pulmonary TB in adolescents and adults is variable, and BCG does not consistently induce sterilizing immunity [3,4]. These limitations highlight the need for vaccines that generate durable and appropriately regulated protective immune responses.

Despite its established role in preventing severe childhood TB, BCG provides variable protection against pulmonary disease in adolescents and adults and does not reliably induce sterilizing immunity [5,6]. Protection may also wane over time or fail to generate sufficiently durable pulmonary and mucosal responses, leaving vaccinated individuals susceptible to *M. tuberculosis* infection and subsequent progression to active disease [5,6,7,8]. BCG efficacy further varies across populations and geographic settings, potentially reflecting differences in exposure to environmental nontuberculous mycobacteria, host genetics, nutritional status, microbial environments, and BCG strain [5,9]. Together, these limitations underscore the need to better understand the immune mechanisms underlying BCG-induced protection and variability.

Beyond its effects on conventional adaptive immunity, BCG has emerged as an important model for understanding trained immunity and the capacity of innate immune cells to develop long-lasting functional changes following vaccination [5,9].

### 1.3. Expanding the Immunological Framework for TB Vaccination

TB vaccination is an example of trained immunity, which reprograms the innate immune system to respond to a similar stimulus it countered before. In addition to antigen-specific adaptive immunity, through epigenetic reprogramming, BCG can constantly modify innate immune responses. The study has demonstrated that induced innate immunity is individual-dependent. Pre-existing host immunity will determine the likelihood of reprogramming results. This could also possibly explain why different individuals respond to BCG heterogeneously [10].

Because TB infection usually starts from the mucosa, mucosal/tissue-resident immunity training is another important aspect against *M. tuberculosis*. Tissue-resident memory T (T_RM_) cells have been indicated in protective immune responses against not only viral infections, but mycobacterial infections. Through experiments, mucosal BCG vaccination is confirmed to generate lung-resident memory T cell populations giving protection against pulmonary TB [11].

In addition, recently the search for correlates of vaccination protection against *M. tuberculosis* has gradually shifted from individual immune markers toward multidimensional approaches. Correlates of protection (CoPs) are designed to evaluate different T-cell/B-cell responses, including the above-mentioned trained innate immunity, and mucosal/tissue-resident immune responses holistically. A reliable CoP can facilitate the down-selection of vaccines during the clinical pipelines, and thus plays an important role in TB vaccine development [12].

## 2. Immunological Foundations of Protection Against *M. tuberculosis*

### 2.1. Early Host–Pathogen Interactions

Early host–pathogen interactions begin when *Mycobacterium tuberculosis* (Mtb) enters the alveoli within the lungs and encounters resident alveolar macrophages. As part of the immune system, these alveolar macrophages engulf the bacterium for elimination but paradoxically become one of its preferred replicative niches [13]. Infected alveolar macrophages relocate within the lung tissue and drive the dissemination process before peripheral neutrophils and monocyte-derived macrophages are recruited [14]. Recent comparative studies demonstrate differing responses between the alveolar and monocyte-derived macrophages within similar granuloma environments, with this heterogeneity pointing to containment or spread of infection. This phenotypic difference offers a target for host-directed intervention [13]. Dendritic cell involvement helps bridge the innate and adaptive immune response, allowing T-cell priming [15]. Dendritic cell presentation is another important step that affects the quality of the immune response, making it targetable for vaccine engineering by conjugating Mtb antigens to a rabies virus glycoprotein (RVG) peptide, which enhances antigen uptake and presentation [16]. Granuloma formation is also crucial for understanding the early immune response to Mtb. While once believed to provide a protective barrier to systemic circulation, the granuloma is now better understood to promote bacterial proliferation by providing a supply of macrophages for Mtb to proliferate and grow [17].

### 2.2. Innate and Adaptive Immune Coordination

**Figure 1:**
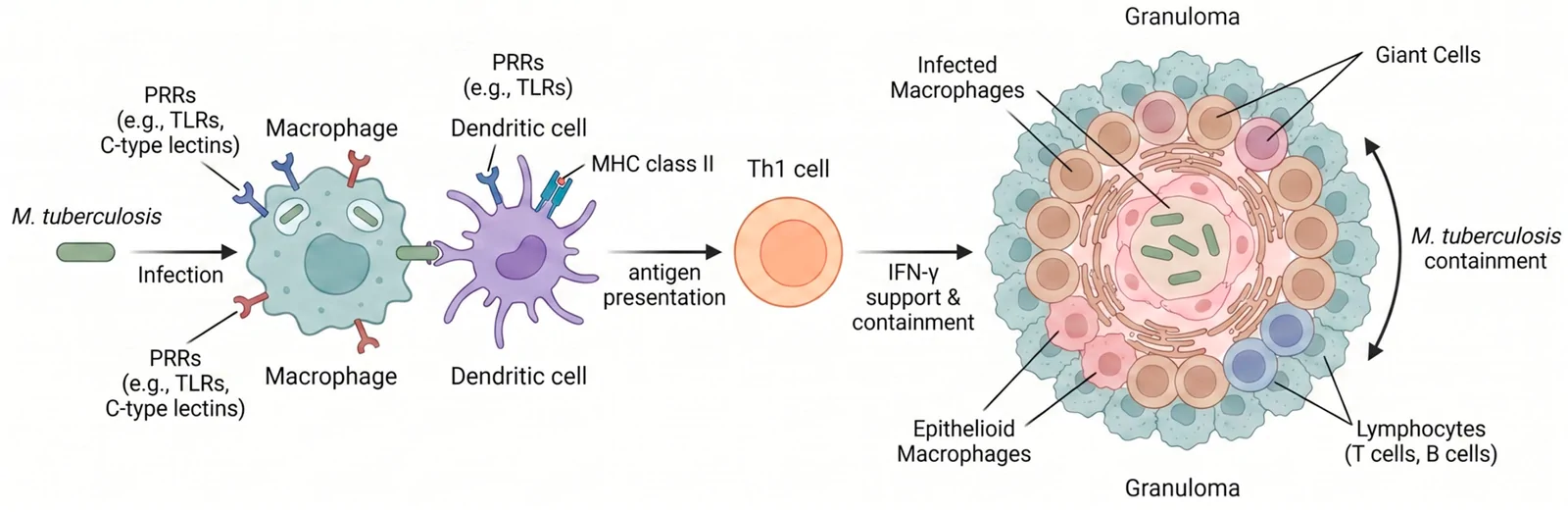
Innate recognition and Th1-mediated granuloma formation during Mycobacterium tuberculosis infection. Following pulmonary infection, *M. tuberculosis* is recognized and internalized by macrophages and dendritic cells through pattern-recognition receptors (PRRs). Dendritic cells present mycobacterial antigens to CD4^+^ T cells, promoting Th1 differentiation and interferon-γ–mediated macrophage activation. These responses drive granuloma formation, characterized by infected and epithelioid macrophages, multinucleated giant cells, and surrounding T and B cells. Created in BioRender. Chacon, J. (2026) https://BioRender.com/x29ve8a, accessed on 5 October 2026.

Innate recognition of *M. tuberculosis* (Mtb) is initiated through pattern-recognition receptors (PRRs) that detect pathogen-associated molecular patterns and activate macrophages, dendritic cells, and inflammatory signaling pathways (Figure 1) [18]. Cytokines are central to coordinating these responses: TNF-α supports granuloma organization, IL-1β promotes inflammation and immune-cell recruitment, and IL-12 drives IFN-γ production required for effective macrophage activation [19,20,21]. Neutrophils and natural killer (NK) cells also contribute to early host responses, although Mtb can exploit neutrophil necrosis to facilitate macrophage infection [22]. NK cells provide additional cytotoxic and cytokine-mediated activity and can exhibit memory-like responses, including enhanced cytokine production following BCG vaccination [23,24].

Adaptive immunity develops more slowly, creating an early window during which Mtb can establish infection before effective T-cell responses reach the lung [25]. CD4^+^ Th1 cells are central to protection through IFN-γ-mediated macrophage activation, while CD8^+^ T cells contribute by recognizing and eliminating infected cells [26,27,28,29]. B cells and antibodies may provide additional protective functions, although their contributions to TB immunity remain less well defined than those of T cells [30]. Importantly, the magnitude of conventional cell-mediated immunity alone does not necessarily predict protection. In the MVA85A trial, vaccine-induced cellular immune responses in BCG-vaccinated infants did not translate into improved protection against TB [31]. Collectively, these findings suggest that effective immunity against Mtb depends on the coordinated activity of innate and adaptive responses rather than on a single immune pathway.

### 2.3. Nonconventional T Cells in M Tuberculosis Host Defense 

In addition to conventional CD4^+^ and CD8^+^ T cells, nonconventional T-cell populations are increasingly recognized as important components of host defense against *Mycobacterium tuberculosis*. These populations include γδ T cells, mucosal-associated invariant T (MAIT) cells, natural killer T (NKT) cells, and other donor-unrestricted T cells (DURTs), which recognize microbial antigens through relatively conserved antigen-presentation pathways rather than classical polymorphic MHC restriction. This property enables rapid, innate-like responses and may be particularly relevant at mucosal sites where *M. tuberculosis* first encounters the host. MAIT cells recognize microbial riboflavin-derived metabolites presented by MR1, whereas NKT cells recognize lipid antigens presented by CD1d. Vγ9Vδ2 γδ T cells can respond rapidly to mycobacterial phosphoantigens and produce cytokines including IFN-γ and TNF-α while exerting cytotoxic activity against infected cells. Collectively, these populations can bridge innate and adaptive immunity through rapid cytokine production, cytotoxicity, and modulation of macrophage function. However, their precise contributions to protective immunity in human TB remain incompletely defined. Their conserved antigen-recognition pathways, rapid effector functions, and enrichment at mucosal sites nevertheless make nonconventional T cells attractive complementary targets for future TB vaccine development. Current vaccine candidates therefore target multiple immune pathways, including conventional antigen-specific CD4^+^ and CD8^+^ T-cell responses, nonconventional T-cell populations, tissue-resident memory cells, trained innate immunity, and functional antibody responses [32,33,34].

### 2.4. Immune Evasion and Implications for Vaccination

Mtb employs multiple, overlapping mechanisms to evade and manipulate host immunity [35]. The bacterium alters macrophage function and inflammatory signaling, promotes progressive T-cell exhaustion, and can enter a metabolically dormant state that facilitates persistence despite host immune and antimicrobial pressures [35,36,37]. Mtb may also modulate host responses through changes in non-coding RNA expression, with distinct microRNA profiles observed in macrophages infected with virulent and avirulent strains [38]. Importantly, attempts to restore T-cell function through immune checkpoint inhibition have been associated with TB reactivation, highlighting the need to balance antimicrobial immunity with immune-mediated pathology [37].

These mechanisms also help explain why generating a strong systemic Th1 response alone may be insufficient for vaccine-mediated protection. Although Th1-derived IFN-γ is essential for macrophage activation and control of Mtb, the magnitude of circulating Th1 responses does not necessarily reflect the quality, localization, or durability of immunity at the pulmonary site of infection. Moreover, Mtb can persist within macrophages and modulate T-cell function despite robust systemic cellular responses. Thus, effective vaccination may require coordinated immunity that extends beyond systemic Th1 responses to include early innate activation, durable immune memory, and appropriately localized responses within the respiratory tract

## 3. Trained Immunity and TB Vaccination

### 3.1. BCG as a Model of Trained Immunity

These constraints necessitate a deeper examination of BCG immunology, particularly the emerging concept of trained immunity. While vaccine efficacy was once viewed almost exclusively through the lens of adaptive immunity, namely antigen-specific T and B cells, BCG has redefined our understanding of innate immune memory [10,39,40]. It is now a primary model for studying the long-term functional reprogramming of monocytes, macrophages, natural killer cells, and hematopoietic progenitor cells [10,39,40].

At its core, BCG-induced trained immunity is driven by epigenetic reprogramming. Exposure to the vaccine alters chromatin accessibility and histone configurations, effectively “priming” antimicrobial genes for rapid activation during subsequent pathogen encounters [10,39]. Unlike traditional adaptive memory, this process is not antigen-specific; rather, it enhances the velocity and intensity of the innate immune response to a broad spectrum of stimuli [10,39].

This innate immune memory has been most extensively characterized in monocytes and macrophages, which, after BCG vaccination, display heightened production of pro-inflammatory cytokines, including TNF-α, IL-1 β, and IL-6, together with phagocytic and microbicidal activity upon re-exposure to both mycobacterial and unrelated stimuli [39]. 

Similarly, in natural killer cells BCG vaccination increases the capacity for IFN-y and pro-inflammatory cytokine secretion following a heterologous stimulation, which is still detectable months after vaccination [24]. Additionally, it is also shown to increase non-specific protection against other pathogens [24].

Experimental studies in humans further demonstrate that BCG vaccination induces persistent transcriptional reprogramming within the hematopoietic stem and progenitor cell compartment, including enrichment of myeloid- and granulocyte-associated programs, providing a mechanistic basis for the durability of trained immunity (see Figures 1, 3 and 4 in Cirovic et al. [41]).

Long-lasting trained immunity is thought to occur upstream at the level of hematopoietic progenitor cells in the bone marrow. Because mature monocytes and natural killer cells are relatively short-lived, BCG- induced chromatin rearrangements in these progenitors stimulate myelopoiesis and give rise to a continuous supply of altered myeloid cells with enhanced antimicrobial capacity. This provides a cellular foundation for trained immunity that can persist for months to years after vaccination [39]. 

### 3.2. Immunometabolic Reprogramming

BCG-induced trained immunity is closely linked to metabolic reprogramming of innate immune cells. Following BCG exposure, monocytes and macrophages undergo shifts in key metabolic pathways, including increased glycolysis and alterations in oxidative phosphorylation and tricarboxylic acid cycle activity. These changes provide both the energy and metabolic intermediates required to support enhanced inflammatory and antimicrobial responses and can interact with epigenetic remodeling to establish a more responsive innate immune state [39,42]. Such mechanisms may help explain observations that BCG vaccination is associated, in certain contexts, with reduced incidence of non-TB respiratory infections and infection-related mortality, effects thought to reflect enhanced innate immune responsiveness rather than antigen-specific protection against unrelated pathogens [43]. 

Importantly, immunometabolic responses to BCG are not uniform across individuals. Human multi-omics studies indicate that baseline immune and epigenetic states can influence the magnitude and characteristics of trained immunity following vaccination [10]. This heterogeneity suggests that variability in BCG-induced protection may partly reflect differences in host factors, including prior microbial exposures, immune state, epigenetic regulation, and metabolic programming. These observations are particularly relevant to TB because *Mycobacterium tuberculosis* can manipulate host metabolic pathways, including glucose and lipid metabolism, to promote intracellular survival and persistence. Immunometabolism therefore represents both a mechanism underlying vaccine-induced immunity and a potential target for improving anti-mycobacterial defense [44,45]. Future vaccine and host-directed strategies may seek to intentionally shape metabolic pathways to enhance macrophage antimicrobial function, strengthen vaccine-induced responses, and support the generation and maintenance of durable immune memory.

### 3.3. Heterologous Effects of BCG

The functional reprogramming described above is not antigen-specific, and its clearest clinical expression is the heterologous, or non-specific, effects attributed to BCG. Epidemiological and trial data have long suggested that BCG vaccination is associated with reductions in all-cause mortality and in non-mycobacterial infections that exceed what protection against TB alone would predict, although effect sizes vary considerably across settings and study designs [43]. Controlled human studies provide more direct mechanistic support: BCG vaccination before experimental challenge with an attenuated yellow fever vaccine virus reduced viremia, and this protection correlated with the capacity of circulating monocytes to produce IL-1β rather than with antigen-specific adaptive responses, consistent with epigenetic and metabolic reprogramming associated with trained immunity [46]. The experimental findings from this human challenge model provide direct evidence linking BCG-induced innate immune reprogramming with heterologous protection (see experimental figures in Arts et al. [46]). Clinical translation has nonetheless been inconsistent: in the ACTIVATE trial, BCG vaccination of older adults at hospital discharge prolonged the time to first infection and reduced the incidence of new infections, particularly those of respiratory viral origin, whereas the BRACE trial found no significant reduction in COVID-19 incidence or severity among BCG-vaccinated health care workers [47,48]. These divergent outcomes reinforce that heterologous protection is context-dependent and modulated by host factors including age, sex, prior mycobacterial exposure, baseline inflammatory state, BCG strain, and the specific pathogen encountered [10]. Even so, these findings establish that a vaccine can measurably alter innate immune function in humans for months to years and that such changes can confer protection against pathogens the vaccine does not encode, supporting the deliberate incorporation of innate immune reprogramming into next-generation vaccine design rather than treating it as an incidental property of mycobacterial vaccination.

### 3.4. Implications for Next-Generation Tuberculosis Vaccines

In summary, the historical and clinical significance of BCG remains profound due to its role in preventing severe childhood tuberculosis. Yet, its limitations, variable adult efficacy, lack of sterilizing immunity, and geographic inconsistency, emphasize the urgent need for next-generation vaccines. Simultaneously, BCG has revolutionized modern immunology by providing a framework for understanding trained immunity, epigenetic reprogramming, and the metabolic foundations of the innate immune system.

## 4. Mucosal and Tissue-Resident Immunity in TB Vaccination

### 4.1. Why the Lung Matters

Mucosal immunity has become one of the most important emerging areas in TB vaccine research because *Mycobacterium tuberculosis* enters the body primarily through inhaled respiratory droplets. This means the first immune battle occurs at the airway and lung mucosa, not in the blood. Traditional TB vaccination strategies, especially intradermal BCG, generate systemic immune responses but may not reliably produce strong, durable immune protection at the lung surface where infection begins [49,50]. This has led researchers to propose that one major weakness in current TB vaccine design is the failure to generate sufficient local mucosal immunity.

Systemic immunity is important, but it may be insufficient for preventing pulmonary TB because circulating immune cells may arrive too late to stop early bacterial establishment. After inhalation, *M. tuberculosis* can infect alveolar macrophages and delay effective adaptive immune activation, giving the bacteria time to replicate before strong T-cell responses reach the lung. Therefore, an ideal TB vaccine should not only produce systemic T-cell immunity, but also position immune memory directly within the respiratory tract [50,51]. This is why mucosal vaccination is being studied as a strategy to generate immune protection at the site of infection rather than relying only on immune cells circulating in the blood.

### 4.2. Tissue-Resident Memory T Cells 

A key component of mucosal protection is the tissue-resident memory T cell (TRM). Unlike central memory or effector memory T cells that circulate through blood and lymphoid organs, TRM cells remain embedded within tissues such as the lung parenchyma and airway mucosa. Their location allows them to respond rapidly when a pathogen enters the same tissue again. In TB, this is especially important because early containment of *M. tuberculosis* in the lung may determine whether infection is cleared, contained, or progresses to active disease [50,51].

Recent TB vaccine studies support the idea that pulmonary or mucosal vaccination can generate stronger lung-localized T-cell responses than systemic vaccination alone. Experimental studies provide direct evidence that the route of BCG administration influences both pulmonary protection and the localization and phenotype of vaccine-induced T-cell responses. In mice, mucosal BCG vaccination enhanced protection compared with systemic vaccination and selectively generated antigen-specific PD-1^+^KLRG1^−^ CD4^+^ T cells within the lung parenchyma and airways (see Figures 3–6 in Bull et al. [52]). Additional experimental work suggests that pulmonary vaccine strategies can stimulate *M. tuberculosis*-specific CD4^+^ TRM cells in the lung, supporting the idea that TRM induction should be considered an important benchmark for future TB vaccine design [51,53].

### 4.3. Mucosal Antibody Responses

Mucosal immunity also includes humoral responses, particularly immunoglobulin A, or IgA. IgA is the major antibody class found at many mucosal surfaces and can contribute to immune exclusion by limiting pathogen attachment, invasion, or spread at epithelial barriers. In TB, antibodies have historically received less attention than T-cell immunity, but this is changing. Although CD4^+^ T helper 1 responses remain central to TB protection, mucosal IgA may provide an additional layer of defense by acting at the airway surface before bacteria fully establish infection [49,54]. This is important because preventing or reducing early bacterial entry at the mucosa could lower the chance of progression to pulmonary disease.

### 4.4. Mucosal Vaccine Delivery

Intranasal vaccines are especially relevant to this discussion because they deliver antigen directly to the respiratory mucosa. Unlike injected vaccines, intranasal vaccines can stimulate local immune responses in the upper and lower respiratory tract, including TRM cells, mucosal B cells, and IgA-producing plasma cells. This approach is attractive for TB because it mimics the natural route of infection and may generate immune memory in the same tissue where *M. tuberculosis* first encounters the host [49,55]. Recent studies in respiratory vaccination more broadly also show that intranasal boosting can convert pre-existing systemic immunity into local mucosal IgA responses, suggesting that systemic prime–mucosal boost strategies may be useful for pathogens that enter through the airway [56].

Pulmonary vaccine delivery is another promising strategy. Aerosolized or inhaled vaccines can target the lower respiratory tract more directly than intranasal administration, potentially inducing immune responses in the lung parenchyma and alveolar spaces where *M. tuberculosis* infection begins. This is particularly relevant because pulmonary TB is the main transmissible form of disease. A recent controlled human study using aerosolized attenuated Mycobacterium bovis BCG showed that inhaled challenge models can characterize early mucosal immune responses in humans, helping researchers better understand which local immune markers may be associated with protection [57]. Animal studies of intranasal BCG have also demonstrated systemic and pulmonary mucosal immune responses, further supporting respiratory delivery as an innovative TB vaccine strategy [58].

### 4.5. Challenges of Mucosal Vaccination

However, mucosal TB vaccination also has important challenges. The lung is a delicate tissue, so vaccines delivered directly to the airway must be safe, noninflammatory, and carefully dosed. Excessive immune activation could worsen lung pathology, while insufficient activation may fail to protect against TB. Delivery is also technically difficult because vaccine particles must reach the correct region of the respiratory tract, remain stable, and induce durable immunity without causing harmful inflammation [49,54]. These challenges explain why mucosal TB vaccines are promising but still require careful clinical evaluation.

### 4.6. Integrating Systemic and Mucosal Immunity

Overall, mucosal immunity may complement systemic immunity by establishing protective responses at the primary site of *M. tuberculosis* entry. Lung-resident memory T cells, mucosal IgA, and local innate and adaptive immune responses may enable more rapid recognition and containment of Mtb following exposure, while systemic immunity provides broader and sustained immune surveillance. Thus, effective TB vaccination may require coordinated local and systemic immunity rather than reliance on either compartment alone. Intranasal and pulmonary vaccine strategies offer promising approaches for achieving this complementary protection and remain important areas of ongoing TB vaccine research.

## 5. Defining Correlates of Protective Immunity

### 5.1. Why TB Lacks a Simple Correlate of Protection

One of the biggest challenges in TB vaccine development is that researchers still do not have a clearly defined correlate of protection (CoP) [12]. A correlate of protection is a measurable immune response that predicts whether an individual is likely to be protected from infection or disease after vaccination [59]. Antibody levels can serve as useful predictors of protection for many vaccines. This is useful because it allows researchers to estimate vaccine effectiveness without waiting for large numbers of disease cases to occur [59]. However, TB is more complex because protection against *Mycobacterium tuberculosis* does not depend on a single immune marker or cell type [12,60]. Evidence suggests that protection involves coordinated interactions among macrophages, T cells, cytokines, lung-resident immune cells, and potentially components of trained innate immunity [12,60].

### 5.2. Limitations of IFN-γ and Conventional Th1 Measurements

Historically, TB vaccine research has focused heavily on Th1 immune responses. More specifically, it has focused on IFN-γ production [60]. IFN-γ is considered a critical cytokine because it activates macrophages, enhancing their ability to restrict bacterial replication [60]. The importance of this pathway is further supported by observations that individuals with genetic defects affecting IFN-γ or IL-12 signaling are highly susceptible to mycobacterial infections [60]. However, IFN-γ alone has not proven to be a reliable correlate of vaccine-induced protection [12]. Several TB vaccine studies have shown that a strong antigen specific IFN-γ or Th1 response does not translate into improved protection. This implies that although IFN-γ is important for immunity, measuring it alone is not sufficient to predict vaccine success [12,60].

### 5.3. Polyfunctional T-Cells

Because IFN-γ has not emerged as a definitive correlate, researchers have investigated the role of polyfunctional T cells [61]. Polyfunctional T cells are capable of simultaneously producing multiple cytokines, including IFN-γ, TNF-α, and interleukin-2 (IL-2) [61]. These cells were proposed as potential correlates because multifunctional immune responses may be effective [61]. Studies have shown associations between polyfunctional CD4^+^ T-cell responses and vaccine-induced immunity, suggesting that these cells may contribute to protection [61]. However, polyfunctional T cells have not consistently predicted protection across vaccine studies and therefore have not been accepted as definitive correlates of protection [61]. One explanation is that immune responses measured in peripheral blood may not accurately reflect immune activity within the lungs, where TB infection is initiated [62]. Additionally, excessive immune activation can contribute to tissue damage and pathology, meaning that stronger immune responses are not always synonymous with better protection [60].

### 5.4. Tissue Localization as a Component of Protection

The location of the immune response presents another major challenge in identifying TB correlates of protection [62]. TB is acquired through inhalation of infectious aerosols, and the earliest interactions between the host immune system and *M. tuberculosis* occur in the respiratory tract and lungs [62]. Most TB vaccine studies rely on blood samples because they are relatively easy to obtain, but blood-based measurements may not accurately represent protective immunity within pulmonary tissues [62]. Evidence suggests that tissue-resident memory T cells located in the airways and lungs may play an important role because they are already positioned at the site of infection and can respond rapidly to invading bacteria [62]. These findings have contributed to growing interest in mucosal TB vaccines designed to generate stronger local immunity in the respiratory tract [62].

### 5.5. Trained Immunity as a Potential Component of Protective Signatures

Because conventional Th1 measurements, polyfunctional T-cell frequencies, and blood-based readouts have each proven insufficient, attention has turned to whether trained innate immunity should be incorporated into protective signatures. BCG-induced reprogramming of monocytes, macrophages, and NK cells enhances cytokine production and antimicrobial function upon restimulation, and these changes are sustained by epigenetic remodeling of hematopoietic progenitors in the bone marrow. In mice, BCG access to the bone marrow compartment reprograms hematopoietic stem cells toward myelopoiesis and generates epigenetically modified macrophages with enhanced capacity to restrict *M. tuberculosis* growth [63]. These experiments provide direct evidence that BCG-mediated reprogramming of hematopoietic stem cells can generate sustained protective innate immunity (see experimental figures in Kaufmann et al. [63]). Comparable progenitor-level changes have subsequently been demonstrated in humans (see Figures 3 and 4 [41]).

Measurements derived from this biology differ fundamentally from antigen-specific assays, assessing functional capacity through ex vivo cytokine production following heterologous stimulation together with chromatin accessibility and immunometabolic profiles [42]. Baseline epigenetic and immune cell states also predict the magnitude of trained immunity induced by vaccination, suggesting that pre-vaccination profiling could identify individuals likely to mount a robust innate response [10]. Important limitations remain, however: trained immunity has not been validated as a correlate of protection against TB disease in humans, assays are not standardized across laboratories, and excessive innate activation may contribute to immunopathology rather than control. Trained immunity is therefore best regarded not as a stand-alone correlate but as one dimension that any adequate protective signature must capture alongside adaptive and tissue-localized responses.

### 5.6. Systems Immunology and Transcriptomic Signatures

Systems immunology has emerged as a promising approach for addressing this challenge [12]. Rather than focusing on a single immune marker such as IFN-γ, systems immunology integrates multiple layers of biological information, including gene expression profiles, cytokine networks, and metabolic pathways [12]. Transcriptomic approaches have been particularly valuable in TB research because they allow investigators to identify gene-expression signatures associated with disease risk and immune protection [64,65]. In a prospective cohort study, Zak et al. identified a 16-gene blood RNA signature that predicted progression to active TB before clinical diagnosis. Such signatures may help identify individuals at high risk of disease and could open the possibility for targeted intervention [65].

### 5.7. Toward Multidimensional Correlates of Protection

Taken together, these observations indicate that no single immune parameter is likely to serve as a correlate of protection for TB. A more realistic framework incorporates multiple dimensions: the magnitude and functional quality of antigen-specific T-cell responses, their anatomical localization, the contribution of trained innate immunity, and humoral functions that extend beyond antibody titer. Evidence that Fc-dependent antibody functions and distinct IgG glycosylation patterns differ between latent infection and active disease supports inclusion of functional humoral readouts in such a framework [66]. Models in which protection is both substantial and reproducible offer the most tractable route to defining these signatures. Intravenous BCG administration in rhesus macaques prevents or markedly reduces infection in most animals, in contrast to the more modest protection afforded by intradermal delivery [66]. Intravenous BCG also produced substantial and sustained recruitment of T cells to the airways (see Figure 1 in Darrah et al. [67]). Experimental comparisons demonstrated striking reductions in pulmonary inflammation, granuloma formation, pathology, and mycobacterial burden following intravenous BCG vaccination (see Figure 2 in Darrah et al. [67]), making this model particularly valuable for identifying immune parameters associated with protection rather than merely with vaccination. Controlled human infection models using attenuated mycobacteria provide a complementary approach for characterizing early mucosal responses in humans [57]. Representative experimental evidence supporting these platform-specific immune responses and protective mechanisms is discussed below, with reference to key preclinical and clinical studies and their associated experimental findings.

A further distinction is important. Correlates of risk, such as the 16-gene blood RNA signature that predicts progression to active disease, identify individuals likely to develop disease but do not necessarily identify the mechanisms by which a vaccine protects [65]. The same applies to the activated HLA-DR^+^ CD4^+^ T-cell phenotype associated with TB risk in BCG-vaccinated infants [68]. Establishing a validated correlate of protection will therefore require prospective evaluation within efficacy trials, harmonized assays, and regulatory consensus on acceptable evidence [59]. A reliable multidimensional correlate would substantially accelerate TB vaccine development by enabling earlier and more rational down-selection of candidates [12]. 

## 6. Applying Immunological Principles to Next-Generation TB Vaccines

**Figure 2:**
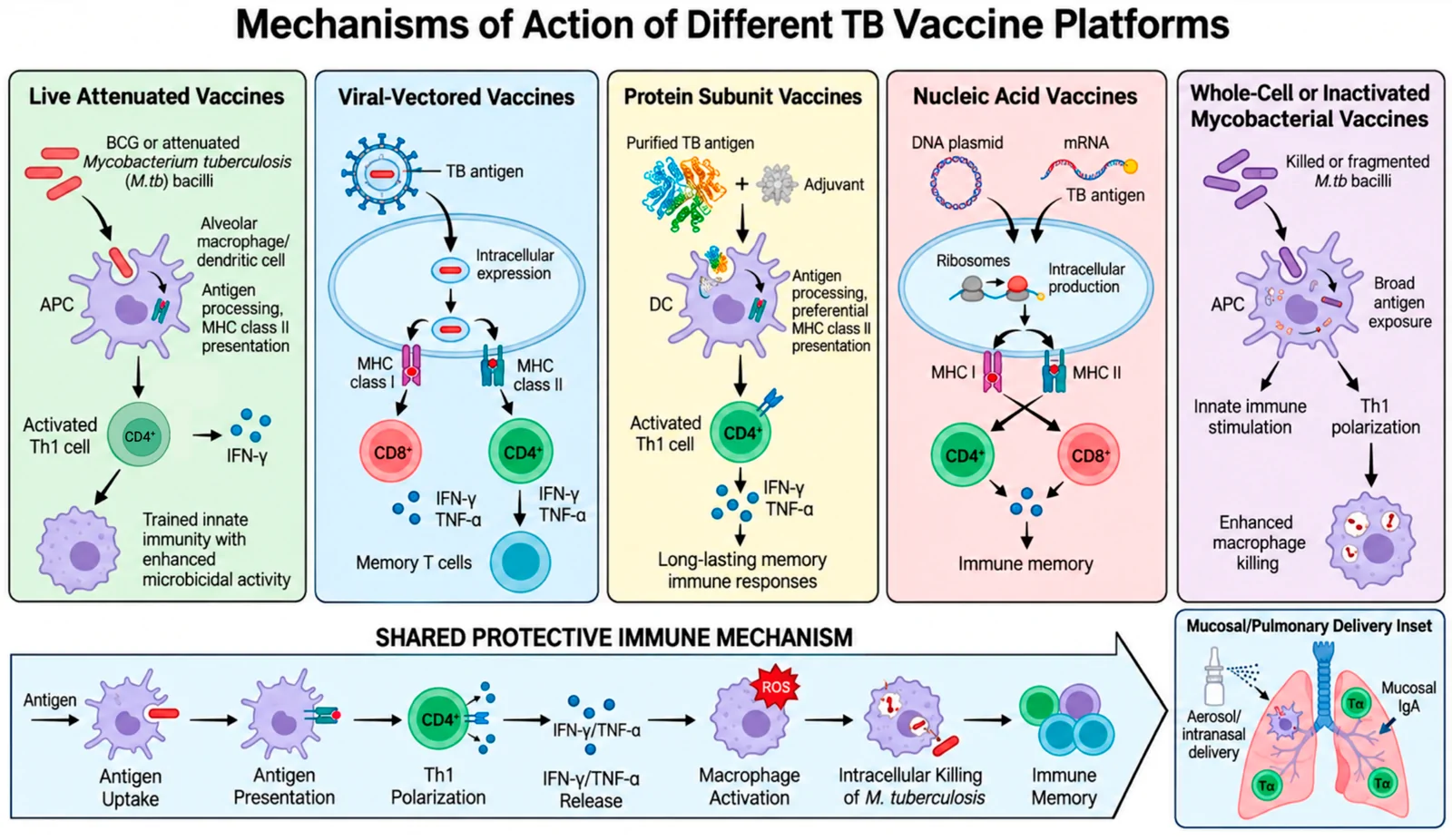
Tuberculosis vaccine platforms, induced immune responses, and potential protective mechanisms. Major tuberculosis vaccine platforms, including live-attenuated, viral-vectored, protein-subunit, nucleic acid, and whole-cell vaccines, use distinct approaches to deliver *Mycobacterium tuberculosis* antigens to the immune system. These platforms induce overlapping but platform-dependent immune responses, including antigen presentation, Th1-polarized CD4^+^ T-cell responses, CD8^+^ T-cell responses, IFN-γ and TNF-α production, antibody responses, and the generation of immune memory. These responses may contribute to protection by enhancing macrophage activation and intracellular mycobacterial control, promoting more rapid recall responses following exposure, and limiting bacterial replication and disease progression. Mucosal vaccine delivery may additionally promote tissue-resident pulmonary immunity and local IgA responses at the site of *M. tuberculosis* entry. Created in BioRender. Chacon, J. (2026) https://BioRender.com/iwxr63j, accessed on 6 October 2026.

Next-generation TB vaccine development increasingly seeks to translate advances in TB immunology into strategies that generate more effective and durable protection. Rather than focusing solely on the magnitude of antigen-specific responses, contemporary approaches aim to influence the quality, breadth, localization, and persistence of immunity. Protein-adjuvant, viral-vectored, live-attenuated, nucleic acid, and whole-cell vaccine platforms use distinct antigen-delivery strategies that shape overlapping but platform-dependent immune responses, including antigen-specific CD4^+^ and CD8^+^ T-cell immunity, Th1 polarization, innate immune activation, antibody responses, immune memory, and potentially tissue-localized immunity (Figure 2). These responses may contribute to protection through enhanced macrophage activation and intracellular control of *Mycobacterium tuberculosis*, more rapid recall responses following exposure, and restriction of bacterial replication and disease progression. Mucosal delivery may further promote tissue-resident pulmonary immunity and local IgA responses at the site of infection. Thus, the distinct immunological properties of these vaccine platforms provide opportunities to address several limitations associated with conventional TB vaccination.

### 6.1. Vaccine Platforms as Tools for Shaping Protective Immunity

Different vaccine platforms engage the immune system through distinct mechanisms. Protein-adjuvant vaccines combine selected *M. tuberculosis* antigens with immunostimulatory adjuvants that enhance innate activation and promote antigen-specific T-cell responses [69]. Viral-vectored vaccines deliver antigen-encoding sequences into host cells, allowing endogenous antigen expression and presentation through MHC pathways and promoting CD4^+^ and CD8^+^ T-cell responses [70,71,72]. Live-attenuated vaccines provide broader antigenic exposure and engage multiple innate and adaptive pathways, including effector, central memory, and tissue-resident memory T-cell populations [73,74,75]. In contrast, mRNA-based platforms offer a flexible means of delivering defined combinations of Mtb antigens and inducing cellular immune responses without administration of a live organism [76,77].

Importantly, these platforms should not be viewed solely as alternative methods of antigen delivery. Their value also lies in their capacity to influence the magnitude, phenotype, localization, and durability of vaccine-induced immunity. Understanding these immunological differences may help identify which combinations of immune responses are most relevant to protection against pulmonary TB.

### 6.2. Selected Vaccine Candidates as Immunological Case Studies

Several vaccine candidates illustrate how these principles are being applied to TB vaccine development and are listed and described below.

M72/AS01E: antigen-specific immunity and the correlate-of-protection challenge. M72/AS01E combines the Mtb32A and Mtb39A antigens with the AS01E adjuvant system and demonstrated approximately 50% efficacy against progression to active pulmonary TB in adults with latent *M. tuberculosis* infection, with protection sustained during follow-up [78]. The candidate demonstrates that a protein-adjuvant platform can provide clinically meaningful protection against pulmonary disease. Importantly, however, its efficacy also illustrates a central challenge in TB vaccinology: measurable vaccine-induced immune responses do not yet provide a definitive correlate capable of explaining or predicting protection. M72/AS01E therefore provides an important model for linking clinical efficacy with efforts to define protective immune signatures.

Adenovirus-based vaccines: enhancing cellular and pulmonary immunity. Adenovirus-based vectors represent an important subset of viral-vectored TB vaccines because their intracellular expression of mycobacterial antigens can induce both CD4^+^ and CD8^+^ T-cell responses. AERAS-402 (Crucell Ad35), a replication-deficient adenovirus serotype 35 vector expressing Ag85A, Ag85B, and TB10.4, has demonstrated the ability to induce polyfunctional CD4^+^ and CD8^+^ T-cell responses following BCG priming, illustrating the potential of adenoviral vectors as heterologous boosters [79].

Other approaches include AdAg85A and the chimpanzee adenovirus-vectored ChAdOx1 85A, which were developed in part to exploit strong cellular immunogenicity while addressing limitations associated with pre-existing immunity to some human adenovirus vectors [71]. Importantly, adenoviral platforms are also being evaluated through respiratory delivery [80]. Aerosol administration provides an opportunity to combine the cellular immunogenicity of viral vectors with localization of vaccine-induced responses to the respiratory tract, potentially generating immune populations positioned closer to the site of *M. tuberculosis* entry. These approaches illustrate how vector selection, antigen composition, prime–boost strategy, and route of administration can be integrated to shape both the magnitude and anatomical distribution of vaccine-induced immunity.

VPM1002 and MTBVAC: broadening immunity through live-attenuated platforms. Live-attenuated approaches seek to retain the broad antigenic and immunostimulatory properties of mycobacterial vaccination while modifying the organism to enhance immune responses or improve vaccine performance. VPM1002 is a recombinant BCG modified through deletion of urease C and expression of Listeria monocytogenes listeriolysin, whereas MTBVAC is derived from genetically attenuated *M. tuberculosis* and retains a broader repertoire of antigens present in human Mtb isolates [74,75]. These approaches illustrate how modification of live vaccines may alter antigen availability and immune activation while maintaining the capacity to engage multiple components of innate and adaptive immunity.

Mucosal BCG: targeting immunity to the site of infection. The route of vaccination represents another means of shaping protective immunity. Because pulmonary infection begins at the respiratory mucosa, mucosal administration of BCG has been investigated as a strategy for positioning vaccine-induced immunity closer to the site of Mtb entry. Preclinical studies have associated mucosal BCG vaccination with enhanced pulmonary protection and the generation of antigen-specific lung tissue-resident CD4^+^ T cells [52]. This approach illustrates an important principle for next-generation vaccination: protection may depend not only on which immune responses are generated, but also on where those responses are established.

mRNA vaccines: flexible antigen delivery and immune optimization. mRNA platforms provide the ability to rapidly modify antigen composition and deliver multiple defined Mtb antigens within a single vaccine. The BNT164a1 and BNT164b1 candidates each encode eight *M. tuberculosis* antigens distributed among four mRNAs and have demonstrated immunogenicity and protective efficacy in rodent models [77]. In experimental *M. tuberculosis* challenge studies, both candidates significantly reduced pulmonary and extrapulmonary bacterial burden (see Figure 5 in Agrawal et al. [77]), while protection was associated with antigen-specific CD8^+^ T cells displaying a memory-precursor phenotype in the lung (see Figure 6 in Agrawal et al. [77]). Although these candidates remain preclinical, their modular design provides opportunities to optimize antigen combinations and vaccine-induced cellular responses as the immune determinants of TB protection become better defined.

RUTI: Therapeutic vaccination and broad antigenic stimulation. RUTI illustrates a complementary approach in which vaccination is explored as an immunotherapeutic strategy rather than solely for prevention of primary infection. RUTI is a liposomal formulation containing detoxified *M. tuberculosis* cell-wall nanofragments generated under stress conditions, providing a broad repertoire of mycobacterial antigens [81]. Its development has focused on therapeutic applications, including adjunctive treatment, shortening of antimicrobial therapy, and prevention of disease recurrence. This strategy is immunologically distinct from vaccines based on a small number of defined antigens because it seeks to expose the immune system to a wider antigenic repertoire, including antigens potentially associated with different physiological states of *M. tuberculosis*. RUTI therefore illustrates how therapeutic vaccination may complement conventional antimicrobial treatment by attempting to redirect or reinforce host immunity against persistent mycobacterial populations. Its continued clinical evaluation also broadens the conceptual role of TB vaccination from prevention alone toward vaccine-mediated immunotherapy.

Collectively, these examples demonstrate that next-generation TB vaccine development involves more than selecting new antigens or delivery platforms. Vaccine composition, adjuvant selection, platform, and route of administration can each influence the quality, breadth, localization, and persistence of the resulting immune response. As the field moves toward a more integrated understanding of trained immunity, tissue-resident responses, and multidimensional correlates of protection, these immunological principles may increasingly guide the selection and evaluation of vaccine platforms rather than relying primarily on the magnitude of conventional systemic immune responses.

## 7. Host-Directed and Immune-Modulating Strategies

### 7.1. Integrating Trained, Adaptive, and Mucosal Immunity

Future TB vaccination strategies may benefit from integrating multiple layers of host defense rather than relying predominantly on systemic adaptive immunity. Although intradermal Bacillus Calmette-Guérin (BCG) vaccination can provide important protection against severe forms of childhood TB, its protection against pulmonary TB is variable, highlighting the need for strategies that generate effective and durable immunity at the respiratory site of *Mycobacterium tuberculosis* exposure. Mucosal vaccination is therefore being investigated as an approach to establish immune responses directly within the respiratory tract, including tissue-resident T cells and local antibody responses that may provide more rapid recognition and control of *M. tuberculosis* following inhalation. Combined systemic and mucosal vaccination strategies may be particularly valuable by coupling circulating immunity with localized pulmonary protection. However, challenges remain, including the identification of safe and effective mucosal adjuvants, optimization of vaccine formulations, and development of reproducible delivery approaches [49]. 

Protection may also be strengthened by coordinating mucosal and adaptive responses with trained innate immunity. BCG and other vaccine platforms can induce functional reprogramming of innate immune cells and their progenitors, potentially allowing enhanced responses to subsequent microbial encounters. In parallel, antigen-specific CD4^+^ and CD8^+^ T cells, B cells, and memory populations provide targeted and durable adaptive responses. Integrating these complementary mechanisms could therefore generate a broader immune environment in which trained innate responses support early pathogen control, mucosal immunity acts at the site of entry, and adaptive immune memory provides antigen-specific recognition and sustained protection.

Importantly, TB vaccination occurs within populations with heterogeneous immunological histories. Individuals may have prior BCG vaccination, environmental mycobacterial exposure, latent *M. tuberculosis* infection, previous TB disease, or repeated exposure to *M. tuberculosis*, each of which may influence subsequent vaccine responses. The concept of hybrid immunity, which gained prominence during the COVID-19 pandemic to describe immunity generated through combinations of infection and vaccination, illustrates how distinct immune exposures can interact to shape the magnitude and quality of subsequent responses [82]. Although this concept cannot be directly extrapolated to TB, understanding how pre-existing and vaccine-induced immunity interact may help guide future vaccination strategies for populations with differing exposure histories [83]. Collectively, approaches that coordinate trained innate immunity, antigen-specific adaptive responses, and localized mucosal immunity may provide a more comprehensive framework for achieving durable protection against pulmonary TB.

### 7.2. Systems Vaccinology and Biomakrer-Guided Vaccine Development

So far, AI is the most promising invention that will transform every aspect of future vaccine development. AI can be utilized from preclinical discovery to manufacturing, clinical development, and regulatory processes. The process and the cost to design vaccine will be much faster and lower with the help of AI. Through big data analysis, AI is very powerful for (1) facilitating antigen discovery; (2) predicting immune response; (3) accelerating vaccine manufacture; and finally, (4) managing vaccine clinical trial design and performance [84].

### 7.3. Precision Vaccinology

Advances in systems biology, multi-omics technologies, and artificial intelligence are enabling a shift from traditional one-size-fits-all vaccination toward more individualized approaches to TB prevention. Vaccine responses can vary considerably among individuals and populations because of factors such as age, genetic background, prior *Mycobacterium tuberculosis* exposure or infection, immune status, microbiome composition, and other host characteristics. Precision vaccinology seeks to account for this heterogeneity by using biological and clinical information to guide vaccine design, selection, and delivery, with the goal of optimizing protective immunity for specific populations or individuals [82,85].

Within this framework, multiple components of vaccination can potentially be tailored, including antigen selection, adjuvant composition, formulation, dosing schedules, and route of administration. For example, adjuvants may be selected or chemically optimized to generate immune responses appropriate for a particular target population, while combinations of antigens and adjuvants can be refined to enhance the magnitude, quality, localization, or durability of immunity. The route of administration may also be selected according to the desired immune response, including strategies designed to promote immunity at the pulmonary site of *M. tuberculosis* exposure [86]. Biomarkers and immune signatures identified through systems-level analyses could further help identify individuals most likely to benefit from particular vaccine strategies while informing approaches for populations with distinct immune phenotypes or risk profiles.

A major challenge, however, is the large number of possible combinations of antigens, adjuvants, formulations, and delivery strategies, which cannot all feasibly progress through conventional clinical development. Human in vitro systems, humanized animal models, and other translational platforms may therefore provide complementary approaches for screening and prioritizing candidate formulations before advancement to clinical trials [87]. Collectively, precision vaccinology provides a framework for integrating host biology with rational vaccine design and may support the development of TB vaccines better suited to the immunological diversity of populations at risk.

### 7.4. Translating Immunological Insights into Vaccine Design

**Figure 3:**
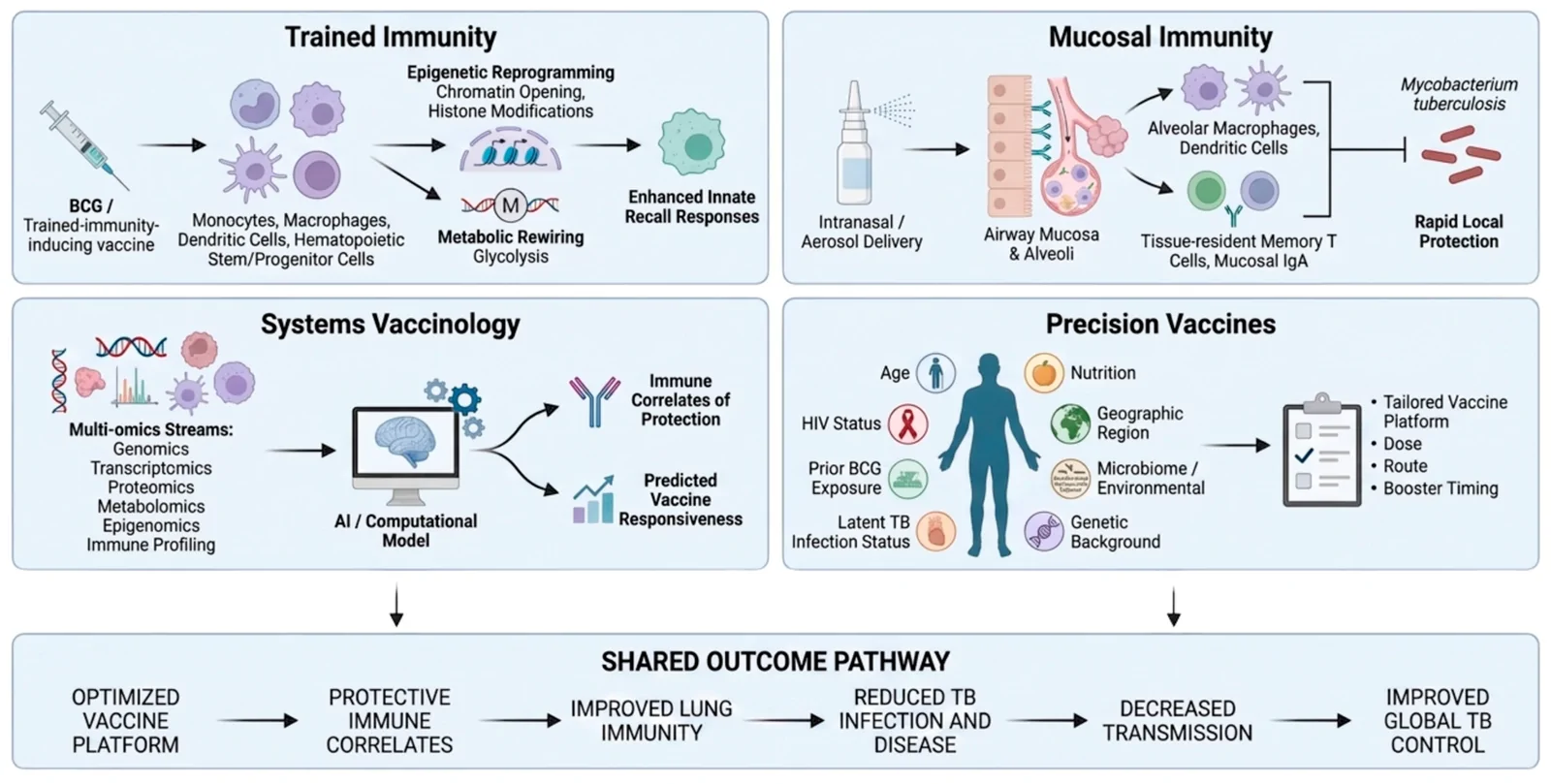
Immune-engineering strategies for next-generation tuberculosis vaccines. Trained immunity, mucosal vaccination, systems vaccinology, and precision-vaccine approaches aim to enhance innate recall, local pulmonary protection, immune-response prediction, and individualized vaccine design. These strategies converge on improved immune correlates, stronger lung immunity, reduced *Mycobacterium tuberculosis* infection and disease, decreased transmission, and improved global tuberculosis control. Created in BioRender. Chacon, J. (2026) https://BioRender.com/xw7sfib. accessed on 6 October 2026.

The principles discussed throughout this review converge on a practical reorientation of TB vaccine development. Rather than optimizing for the magnitude of systemic antigen-specific responses, vaccine design increasingly treats the quality, breadth, localization, and durability of immunity as the primary objectives. Each element of vaccine construction can be directed toward these ends: platform selection shapes the balance of CD4^+^ and CD8^+^ responses and the degree of innate activation, antigen composition determines breadth, adjuvant choice directs response polarization and durability, and route of administration and prime–boost scheduling determine where immune memory is established and how long it persists (Figure 3).

Realizing this approach depends on resolving the evaluation bottleneck. Without a validated correlate of protection, candidates must advance to large and costly efficacy trials to be assessed, which limits how many design variations can be tested [88]. Controlled human infection models, high-protection animal models, and systems-level immune profiling therefore serve a dual function, generating mechanistic insight while providing the comparative readouts needed to prioritize candidates before clinical advancement, ideally aligned with WHO preferred product characteristics so that optimization remains directed toward clinically meaningful endpoints [89]. Immunological insight, evaluation strategy, and implementation planning are thus interdependent components of TB vaccine development rather than sequential stages.

## 8. Conclusions

More than a century after the introduction of Bacillus Calmette-Guérin (BCG), tuberculosis remains a major global infectious disease, reflecting both the complex immune-evasion strategies of *Mycobacterium tuberculosis* and the limited and variable protection provided by existing vaccination approaches. Effective and durable protection will likely require not only robust pathogen-specific responses but also coordinated engagement of innate, adaptive, and tissue-localized immunity. Advances in systems immunology and host–pathogen biology are increasingly informing strategies designed to achieve this goal, including modulation of T- and B-cell responses, multi-epitope vaccine platforms, combined mucosal and systemic immunization, host-directed immunomodulation, recombinant technologies, precision vaccinology, and the application of artificial intelligence and computational biology to vaccine discovery and optimization. Collectively, these approaches represent a shift from antigen delivery alone toward rational engineering of the quality, localization, and durability of protective immune responses. Continued integration of basic, translational, and clinical research will be essential for translating these immunological advances into safer and more effective vaccines and for advancing global efforts toward the WHO goal of ending the TB epidemic.

## Data Availability

No new data were created or analyzed in this study. Data sharing is not applicable to this article.

## Abbreviations

Ag85A	Antigen 85A
AI	Artificial Intelligence
APC	Antigen Presenting Cell
AS01E	Adjuvant System 01E
BCG	Bacille Calmette-Guérin
CoP	Correlate of Protection
COVID-19	Coronavirus Disease 2019
CTLA-4	Cytotoxic T-Lymphocyte-Associated Protein-4
DNA	Deoxyribonucleic acid
ESX-1	ESAT-6 secretion system 1 (type VII secretion system 1)
HIV	Human Immunodeficiency Virus
IFN-γ	Interferon-Gamma
IgA	Immunoglobulin A
IL-1β	Interleukin-1 Beta
IL-2	Interleukin-2
IL-12	Interleukin-12
LNP	Lipid Nanoparticle
M72/AS01E	M72 recombinant fusion protein vaccine formulated with the AS01E adjuvant
MBOVAC 1.0	Multi-epitope vaccine candidate against bovine tuberculosis
MHC	Major Histocompatibility Complex
mRNA	messenger Ribonucleic Acid
Mtb	*Mycobacterium tuberculosis*
MTBVAC	live-attenuated Mycobacterium Tuberculosis Vaccine Candidate
MVA85A	Modified Vaccinia Ankara Vector Expressing Antigen 85A
NK	Natural Killer
NLRP3	NLR family pyrin domain-containing Protein 3
PAMP	Pathogen-Associated Molecular Pattern
PD-1	Programmed cell Death protein 1
PD-L1	Programmed Death-Ligand 1
PRR	Pattern-Recognition Receptor
rBCG	Recombinant Bacille Calmette–Guérin
RNA	Ribonucleic Acid
RVG	Rabies Virus Glycoprotein
TB	Tuberculosis
TCM	T Central Memory cell
TEM	T Effector Memory cell
TLR	Toll-Like Receptor
TRM	Tissue Resident Memory T cell
WHO	World Health Organization
